# Mycetoma: A Narrative Review of Its Clinical and Epidemiological Spectrum With a Focus on Iran

**DOI:** 10.1002/hsr2.73496

**Published:** 2026-11-10

**Authors:** Shabnam Rezaei, Khadijeh Jaberi, Maryam Meskini, Seyed Davar Siadat, Abolfazl Fateh

**Affiliations:** ^1^ Department of Pathobiology, School of Public Health Tehran University of Medical Sciences Tehran Iran; ^2^ Department of Biology, Qo. C. Islamic Azad University Qom Iran; ^3^ Department of Mycobacteriology and Pulmonary Research Pasteur Institute of Iran Tehran Iran; ^4^ Microbiology Research Center (MRC) Pasteur Institute of Iran Tehran Iran

**Keywords:** actinomycetoma, eumycetoma, Iran, Mycetoma, neglected tropical disease

## Abstract

**Background and Aims:**

Mycetoma is a chronic, neglected tropical disease affecting the skin and subcutaneous tissues, capable of extending to deeper structures. It exists in two forms: eumycetoma (fungal) and actinomycetoma (bacterial). This review aims to examine the epidemiology, causative organisms, pathogenesis, clinical features, diagnosis, and management of mycetoma, with a specific focus on the situation in Iran. It also seeks to summarize pathogen‐directed therapies and evaluate the consistency of published Iranian case data.

**Methods:**

This is a narrative review of the literature concerning mycetoma, synthesizing global data and published Iranian case series. The review analyzes information on disease classification, causative pathogens, including *Actinomadura madurae* and *Nocardia* species, clinical presentation, diagnostic methods, and treatment outcomes.

**Results:**

Globally, the relative frequency of actinomycetoma and eumycetoma varies by region. In Iran, mycetoma is reported sporadically, with actinomycetoma predominating, although its reported proportion varies considerably among Iranian series (approximately 54%–85%). The disease most commonly affects the feet of adult men in rural agricultural communities. Diagnosis relies on clinical presentation and grain examination via microscopy, histopathology, and culture. Treatment is pathogen‐directed: actinomycetoma typically responds to prolonged antimicrobial therapy (e.g., co‐trimoxazole and amikacin), while eumycetoma often requires prolonged antifungal therapy (e.g., itraconazole) and surgery, with less successful outcomes due to poor drug penetration.

**Conclusion:**

Greater clinical awareness, earlier diagnosis, and appropriate pathogen‐directed treatment are critical to prevent disease progression, disability, and amputation in mycetoma patients. Preventive measures remain limited, primarily focusing on reducing exposure to contaminated soil. The review highlights the need for more consistent data reporting in Iran to better understand the disease burden and improve management strategies.

## 1 Introduction

Mycetoma is a chronic, progressive infection of the skin and subcutaneous tissues that can extend to deeper structures and cause substantial disability. It is characterized by the classical triad of painless swelling, sinus tract formation, and discharge containing grains. The disease most often affects the lower limbs, particularly the foot, although extrapedal involvement can occur and may be associated with considerable morbidity [1]. Mycetoma develops after traumatic inoculation of environmental organisms and occurs in two forms: eumycetoma, caused by true fungi, and actinomycetoma, caused by aerobic filamentous bacteria [2].

Mycetoma occurs mainly in tropical and subtropical regions, with its distribution influenced by climate, occupation, and environmental exposure. Most causative organisms are soil‐associated and may also be recovered from decaying vegetation, plant material, and thorns. Infection is thought to depend on several factors, including the nature and size of the inoculum and host susceptibility. The higher frequency of disease among men has also been reported consistently, although the reasons for this sex difference are not fully understood. The relative frequency of actinomycetoma and eumycetoma varies geographically. Eumycetoma is particularly common in parts of Africa, whereas actinomycetoma is frequently reported in South and Central America and in other endemic regions [3, 4].

The causative organisms are diverse. Actinomycetoma is associated mainly with *Actinomadura*, *Nocardia*, and *Streptomyces* species, whereas eumycetoma is caused by a broad range of filamentous fungi, including *Madurella*, *Fusarium*, *Aspergillus*, *Curvularia*, and *Pseudallescheria* species. In Iran, the available literature suggests a different pattern from that reported in several other endemic regions, as detailed in Section 2.4 below [5, 6].

Accurate distinction between the two forms is clinically important because their treatment differs substantially. Diagnosis is based on the clinical presentation and examination of grains by direct microscopy, histopathology, and culture. Molecular methods can provide species‐level identification when conventional approaches are inconclusive or unavailable, although access to these techniques remains limited in many endemic settings [7]. Actinomycetoma is generally treated with prolonged antimicrobial therapy, often involving combinations such as trimethoprim‐sulfamethoxazole and amikacin. Eumycetoma is more difficult to treat and usually requires prolonged antifungal therapy, particularly with itraconazole, together with surgical excision when feasible. Advanced disease may involve bone and require more extensive surgery, including amputation in selected cases [8].

Although several reviews have described the global epidemiology and management of mycetoma, the Iranian evidence has not been examined in comparable detail. Published Iranian data are scattered across case reports, case series, and secondary reviews, with substantial variation in reported case numbers, causative organisms, and clinical characteristics. Some larger reviews also appear to include patients described in earlier reports, making direct aggregation of case numbers problematic. This review therefore focuses specifically on the primary Iranian literature and critically evaluates the available epidemiological and clinical data. We also summarize the reported causative organisms, diagnostic approaches, and treatment patterns to provide a clearer picture of mycetoma in Iran and to identify priorities for clinical practice, surveillance, and future research.

## 2 Methods

This narrative review used a structured search of PubMed, Scopus, Web of Science, and Google Scholar. The search was conducted in July 2026 and included literature published from database inception through July 31, 2026. The search included the terms “mycetoma,” “actinomycetoma,” “eumycetoma,” “Iran,” “epidemiology,” “diagnosis,” and “treatment,” alone and in relevant combinations. Eligible studies included primary case reports and case series, original studies reporting epidemiological data from Iran, and recent international guidelines and therapeutic studies relevant to mycetoma management. Secondary reviews were not used as the primary source for Iranian case counts, although they were consulted for background and to identify potentially relevant primary reports.

The available evidence was synthesized across studies, with particular attention to the distribution of causative organisms, demographic and clinical characteristics, geographic patterns, diagnostic approaches, and treatment outcomes. Because several published series may include patients described in earlier reports, case numbers were compared across studies rather than summed to generate a single national total. This was a narrative review, and no formal risk‐of‐bias or quality‐appraisal tool was applied. Study selection was based on the relevance of the available evidence to the review objectives rather than on a preregistered systematic‐review protocol. This approach was considered appropriate because the Iranian literature is dominated by case reports and small, heterogeneous case series, with limited prospective data and potential overlap between reported patient populations. The findings should therefore be interpreted as a critical synthesis of the available evidence rather than as a quantitative estimate of the national burden of mycetoma.

### 2.1 Global Epidemiology of Mycetoma

Mycetoma disproportionately affects people living in rural and economically disadvantaged communities, particularly in areas where access to healthcare, trained personnel, diagnostic services, and effective treatment is limited [8]. These challenges have contributed to the recognition of mycetoma by the World Health Organization (WHO) as a neglected tropical disease [9]. The WHO 2021–2030 NTD roadmap identifies mycetoma as a priority for improved surveillance, development of rapid diagnostic tools, and standardized approaches to field diagnosis and treatment [10]. Although transmission is concentrated in specific endemic regions, cases have been reported from many countries outside these areas, including Israel, Germany, the Netherlands, Turkey, Lebanon, Saudi Arabia, Iran, the Philippines, Japan, Sri Lanka, and Thailand (Table 1) [11, 12, 13].

**Table 1:** Illustrative reported burden of eumycetoma by WHO region based on available country‐level data [8].

WHO region	Representative countries (cases discussed above)	Eumycetoma proportion	Dominant pattern
African region (AFRO)	Chad, Niger, Mali, Mauritania, Togo	26%–95% (wide intra‐regional spread)	Eumycetoma‐dominant overall, but highly heterogeneous
Eastern Mediterranean region (EMRO)	Sudan, Iran	17% (Iran) to 73% (Sudan)	Divergent: Sudan eumycetoma‐dominant, Iran actinomycetoma‐dominant
Region of the Americas (AMRO/PAHO)	Mexico, Brazil (regional data)	3%–62% depending on locality	Actinomycetoma‐dominant nationally in Mexico; intra‐country variation in Brazil
South‐East Asia region (SEARO)	India	42%	Mixed, no strong dominance

The global distribution and relative frequency of eumycetoma and actinomycetoma remain incompletely characterized. Available estimates suggest that eumycetoma accounts for approximately 40% of cases and actinomycetoma for about 60%, although these proportions vary considerably between regions and study populations [14]. Eumycetoma is particularly common in arid regions of Africa, with Sudan representing one of the best‐recognized endemic areas. Environmental conditions characterized by seasonal rainfall and prolonged dry periods may contribute to transmission and persistence of the causative organisms. In these settings, *Madurella mycetomatis* is the predominant eumycetoma pathogen and has been reported to cause more than 70% of cases in some endemic areas [8].

The burden of eumycetoma in Africa remains difficult to quantify because of limited awareness, incomplete surveillance, and restricted access to diagnostic services [15]. Sudan illustrates this problem particularly well. The Mycetoma Research Center has reported more than 12,000 cases through 2024, although this figure represents patients identified through the available healthcare and referral system rather than a population‐based estimate [16]. In Turkana, Kenya, where eumycetoma is common, the annual prevalence of surgically treated mycetoma was estimated at 1.5 cases per 100,000 population [17]. In Senegal, surveillance from three medical centers identified at least 91 eumycetoma cases over a 10‐year period, while the lack of active case‐finding suggests that many cases may have remained undiagnosed [18]. Similarly, 118 cases were reported over 3 years in Ethiopia, but the data came exclusively from referral hospitals and therefore are unlikely to represent the national burden [19]. Reports from Mauritania, Mali, and Chad further demonstrate the presence of eumycetoma in the region, but most available studies are based on single‐ or limited‐center data. Population‐based surveillance remains scarce [18].

Actinomycetoma is reported more frequently in several hot and humid regions, particularly in South and Central America [4]. Mexico provides a clear example, with actinomycetoma accounting for the large majority of reported cases and *Nocardia* species and *Actinomadura madurae* among the principal causative organisms [11, 20]. Across endemic regions, mycetoma predominantly affects men, with reported male‐to‐female ratios commonly ranging from 3:1 to 5:1. Although the disease can occur at any age, patients are often young or middle‐aged adults, many of whom work in farming, herding, or other outdoor occupations involving frequent contact with soil and plant material [21, 22]. Poor socioeconomic conditions and limited access to healthcare may further contribute to delayed diagnosis. Other factors, including malnutrition, diabetes, and impaired immunity, have also been reported in association with mycetoma, although their specific contribution to disease susceptibility requires further study [23, 24].

Available epidemiological data are limited by underreporting, incomplete surveillance, and misdiagnosis, and the reported global burden is therefore likely to underestimate the true extent of disease. Figure 1 summarizes the reported proportions of eumycetoma and actinomycetoma in countries for which more than 10 cases were available. Countries are shown in blue when eumycetoma accounts for a larger proportion of reported cases, in pink when actinomycetoma predominates, and in white when the available data are insufficient to determine the etiological distribution. The available data show substantial geographic variation. For example, two case series from Togo reported 33 cases, including 24 fungal cases, and a further 30 cases without specification of the etiological type. Based on the series with available etiological information, eumycetoma accounted for 24 of 33 cases (73%). Marked differences are also apparent between countries, with eumycetoma representing approximately 17% of reported cases in Iran compared with 95% in Chad. Overall, actinomycetoma appears to predominate across much of the Americas, the Middle East, and the Far East, whereas eumycetoma is more frequently reported in parts of Central Africa. Variation is also evident among countries with relatively large numbers of reported cases. Eumycetoma accounted for approximately 3% of cases in Mexico, 73% in Sudan, and 42% in India. Differences between neighboring countries are similarly striking, including Chad (95%) versus Niger (26%) and Mali (44%) versus Mauritania (83%). Within‐country variation has also been reported. Among 235 cases from Brazil, for example, the proportion of eumycetoma was 62% in Rio de Janeiro, compared with 30% in São Paulo and 33% in Paraná [8].

**Figure 1:**
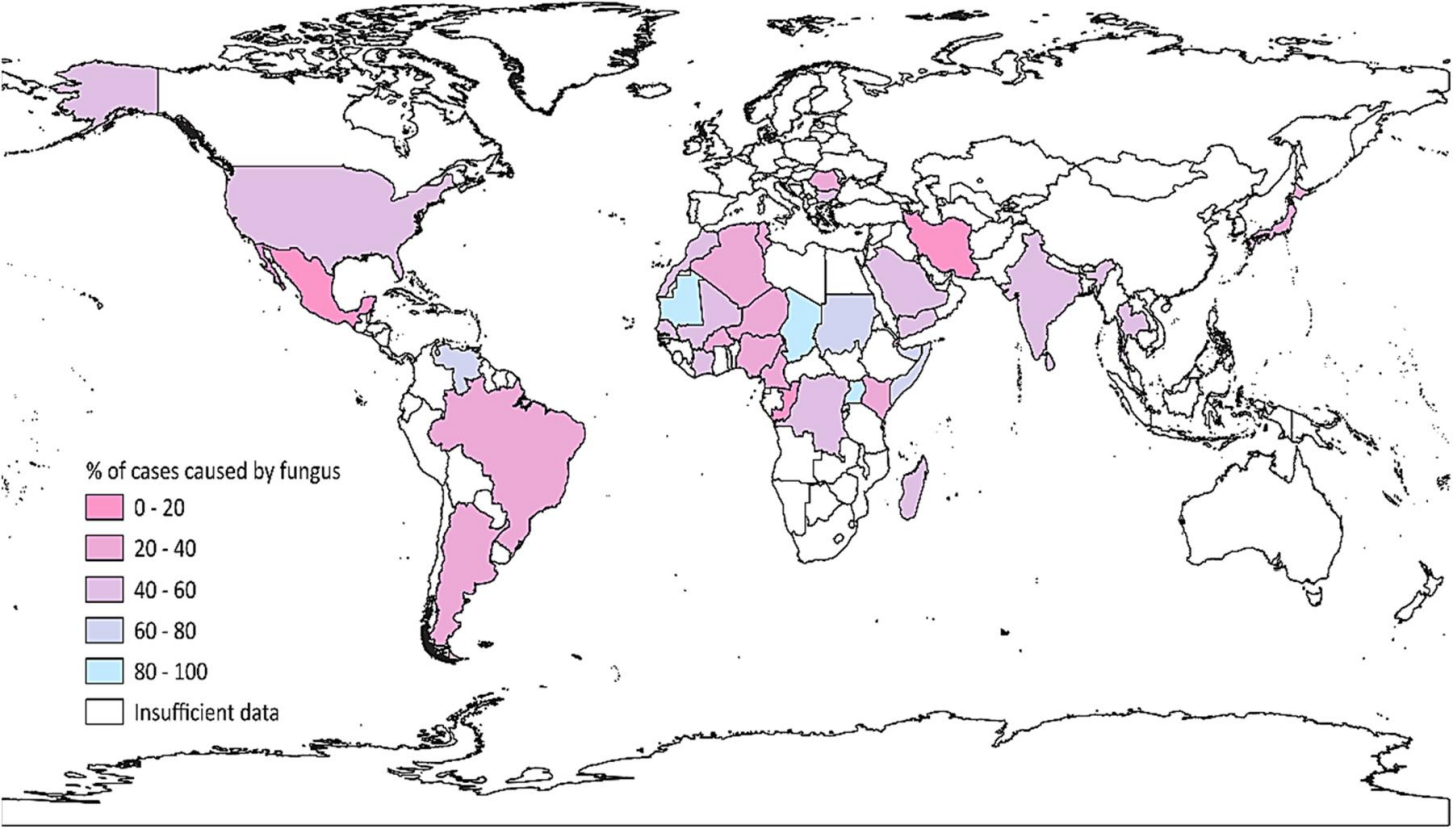
Global distribution of reported actinomycetoma and eumycetoma by country. The map shows the proportion of reported cases attributed to eumycetoma and actinomycetoma in countries with available etiological data. Blue shading indicates a higher proportion of eumycetoma, whereas pink shading indicates a higher proportion of actinomycetoma. White indicates insufficient information to determine the etiological distribution. Data are based on reported cases and should not be interpreted as population‐based estimates of disease burden [8].

### 2.2 Mycetoma in Iran

In Iran, mycetoma is an uncommon but clinically important infection that has been reported sporadically across several provinces. The disease can involve the skin and subcutaneous tissues and, when diagnosis is delayed, may extend to the fascia and bone [25]. As elsewhere, Iranian cases include both actinomycetoma, caused by aerobic filamentous bacteria, and eumycetoma, caused by true fungi [26, 27]. The first documented Iranian cases were reported by Asgari and Alilou in 1972, who described eight mycologically confirmed patients [28]. Since then, additional case reports, case series, and retrospective studies have expanded the available evidence on the geographic distribution, causative organisms, clinical presentation, diagnosis, and treatment of mycetoma in Iran [5, 6, 29, 30, 31]. However, the Iranian literature remains fragmented, and differences in case ascertainment, diagnostic methods, and study populations make the overall epidemiological picture difficult to define.

### 2.3 Epidemiological Features

Available Iranian studies suggest that mycetoma is uncommon and likely underdiagnosed. In the most methodologically detailed series, only 35 of 168 patients referred with suspected mycetoma between 1994 and 2009 were laboratory‐confirmed, corresponding to 20.8%. This finding highlights the difficulty of establishing the diagnosis on clinical grounds alone. A separate nationwide review identified 76 cases reported over 33 years, from 1972 to 2005, across different regions of Iran [29]. Despite differences in study size and duration, the demographic pattern is relatively consistent, with a clear male predominance. Men represented approximately two‐thirds of reported patients in several Iranian series: 66% of 90 cases in one nationwide review, a male‐to‐female ratio of 5:3 in another series, and approximately 67% in the 76‐case review [5, 6, 29]. The predominance among men may partly reflect greater occupational exposure to soil, vegetation, and minor trauma during agricultural and other outdoor activities [32].

Mycetoma can occur from adolescence through older adulthood, but most reported cases occur during the economically active years. In one large review, approximately 60% of patients were aged 31–50 years [6], whereas another study found that 68.5% were older than 40 years [5]. Farmers constituted the largest occupational group, accounting for approximately 44% of cases, followed by housewives (22%), workers, drivers, and military personnel [6, 30]. The occurrence among housewives, including women living in urban areas, suggests that exposure is not limited to agricultural occupations and may also occur through domestic or environmental contact with soil and plant material [29].

The foot was the predominant site of infection, accounting for approximately 74% of reported cases, followed by the leg and arm, each representing about 5% [6]. Other sites included the hand, thigh, buttocks, and head [29]. This distribution is consistent with traumatic inoculation of soil‐ and plant‐associated organisms through minor skin injuries. Barefoot walking and agricultural activities may increase this risk, particularly in rural populations [33]. Disease duration varied considerably, from several months to several decades. Although many patients were diagnosed within 2–3 years of symptom onset, delays of 20 years or more have also been reported [5, 29].

Reported cases have been described mainly in northern, southern, and central Iran. Earlier analyses attributed approximately 36% of cases to the northern provinces of Gilan, Mazandaran, and Golestan and about 27% to southern provinces including Khuzestan, Bushehr, and Bandar Abbas [6, 29]. Other studies have reported a greater concentration in southern and central provinces, with southern regions accounting for approximately 57% of cases [30]. Within northern Iran, Mazandaran accounted for approximately 59% of reported cases from that region.

These geographic patterns should be interpreted cautiously because the available data come from published case reports and series rather than a national surveillance system. Regional differences in case detection, referral patterns, diagnostic capacity, and publication may therefore contribute to the apparent concentration of cases in northern and southern provinces. Whether these patterns also reflect genuine differences in environmental exposure or pathogen distribution cannot be determined from the existing retrospective data. Prospective, population‐based surveillance would be required to address this question [6].

Environmental conditions may nevertheless contribute to regional variation. Warm and humid climates, agricultural activity, and frequent contact with soil and plant material could favor exposure to mycetoma‐associated organisms [5, 34]. Environmental studies from Gilan and Mazandaran have identified organisms associated with human mycetoma, including *Streptomyces griseus*, *Nocardia asteroides*, *Actinomadura madurae*, and *Pseudallescheria boydii*, in soil and plant material. These findings support the presence of potential environmental reservoirs in northern Iran [35].

### 2.4 Causative Agents

Actinomycetoma appears to predominate over eumycetoma among reported Iranian cases, although the estimated proportion varies substantially between studies. It ranged from 54.3% in a hospital‐based series of laboratory‐confirmed cases from 1994 to 2009 to approximately 85% in two large nationwide reviews covering 1972–2005 and 1972–2009, respectively. These differences are more likely to reflect variation in case ascertainment, referral patterns, diagnostic practices, and study periods than a genuine change in the epidemiology of mycetoma. Importantly, the nationwide reviews are not fully independent because some of the later reports include cases described in earlier publications. The reported proportions should therefore not be interpreted as independent estimates of the national distribution of actinomycetoma and eumycetoma [6, 29].

Among the bacterial agents, *Actinomadura madurae* is one of the most frequently reported pathogens and accounted for approximately 24% of all cases in one national review [6]. Other reported actinomycetoma agents included *Nocardia asteroides* (approximately 21%), *Nocardia caviae* (13%), *Actinomyces israelii* (9%), *Nocardia brasiliensis* (4%), and *Streptomyces somaliensis* (2%) [6, 29]. The reported distribution also differs geographically. *A. madurae* has been frequently identified among cases from northern Iran, accounting for approximately 48% in some northern series, whereas *Nocardia* species have been reported more often in southern regions [6]. Given the retrospective and geographically uneven nature of the available data, however, these regional patterns should be regarded as descriptive rather than representative estimates of pathogen distribution across Iran.

Eumycetoma accounts for a smaller proportion of reported Iranian cases, generally less than 15%–16% in the larger reviews [6, 29]. *Pseudallescheria boydii* is among the principal fungal agents reported in Iran and accounted for approximately 10% of all cases in one review. Other reported organisms include *Madurella mycetomatis*, *Aspergillus flavus*, *Aspergillus fumigatus*, and *Fusarium* species [6, 31]. Although relatively uncommon, *A. flavus* eumycetoma has also been documented in Iran. One 2022 case involved a 38‐year‐old male farmer from Khuzestan with type 2 diabetes who developed foot mycetoma after barefoot trauma while working on a rice farm. Molecular identification by β‐tubulin gene sequencing confirmed *A. flavus* [36].

Environmental findings provide additional context for these clinical observations. Mycetoma‐associated organisms have been recovered from soil and plant material in northern Iran, including *Streptomyces griseus*, *Nocardia asteroides*, *Actinomadura madurae*, and *Pseudallescheria boydii* [35]. The detection of *P. boydii* in both environmental and clinical samples supports the plausibility of environmental exposure as a source of infection and is consistent with the strong representation of agricultural workers among reported Iranian patients. However, environmental recovery alone does not establish a direct transmission pathway or quantify the contribution of specific environmental sources to human disease.

### 2.5 Clinical Presentation and Diagnosis in Iranian Patients

The clinical features reported in Iranian patients are broadly consistent with the classical presentation of mycetoma. Infection usually follows minor trauma and initially appears as a painless subcutaneous nodule. The lesion may enlarge slowly over months or years, eventually developing multiple sinus tracts and discharge containing grains [31, 37]. Pain may remain minimal despite progressive disease, allowing infection to extend into the subcutaneous tissues, fascia, and, in advanced cases, bone. Delayed recognition and diagnosis therefore contribute substantially to the morbidity associated with the disease in Iran [29].

Diagnosis requires integration of clinical findings with laboratory and imaging studies. Direct examination of discharged grains can provide useful preliminary information, with fungal hyphae generally observed in eumycetoma and fine, branching bacterial filaments in actinomycetoma [28, 36]. Culture remains an important method for confirming the etiological agent and may be performed on media including Sabouraud dextrose agar, blood agar, and brain–heart infusion agar. Because many causative organisms grow slowly, cultures may require prolonged incubation, sometimes for 6–8 weeks [29]. Histopathological examination, including hematoxylin and eosin and periodic acid–Schiff staining, can demonstrate grains and provide additional evidence of the infectious etiology [30].

Molecular identification is particularly useful when culture or morphological examination does not provide a definitive species‐level diagnosis. In reported Iranian cases, PCR followed by sequencing of fungal targets such as the internal transcribed spacer and β‐tubulin regions has been used to identify unusual or difficult‐to‐characterize isolates, including *A. flavus* [36]. Imaging is important for assessing disease extent, particularly when deeper tissue or bone involvement is suspected. Plain radiography, computed tomography, and magnetic resonance imaging can help define the extent of osseous and soft‐tissue disease and may influence surgical planning and treatment selection [33, 36]. Overall, the Iranian literature supports a stepwise diagnostic approach beginning with clinical assessment and grain examination, followed by culture and histopathology and, where available, molecular identification for species confirmation.

### 2.6 Treatment of Iranian Cases

Treatment of mycetoma in Iran generally follows the same principles used elsewhere and depends on the causative organism, extent of disease, and presence of bone involvement [29]. Actinomycetoma is generally more responsive to antimicrobial therapy than eumycetoma. Reported treatment regimens for actinomycetoma have included trimethoprim–sulfamethoxazole alone or in combination with agents such as streptomycin, dapsone, or amikacin [29, 38]. For eumycetoma, treatment is more challenging and often involves prolonged antifungal therapy with agents such as itraconazole, ketoconazole, or voriconazole, with surgical excision or debridement used when lesions are localized and amenable to surgery [36, 39].

The reported Iranian case of *A. flavus* eumycetoma provides an example of the potential usefulness of species identification and antifungal susceptibility testing in treatment selection. The patient initially received itraconazole at 200 mg/day for 1 month without clinical improvement. Susceptibility testing showed low minimum inhibitory concentrations for voriconazole (0.063 mg/L), posaconazole (0.125 mg/L), itraconazole (0.125 mg/L), and caspofungin (0.5 mg/L), while the MIC for amphotericin B was 1.0 mg/L. Treatment was subsequently changed to voriconazole 200 mg twice daily, with substantial clinical improvement after 2 months, including resolution of purulent discharge, reduction in swelling, and improved mobility [36]. Although this represents a single case, it illustrates how species‐level identification and susceptibility testing may help guide treatment when eumycetoma does not respond to initial therapy.

The Iranian literature overall remains limited by the small number of reported cases and the absence of systematic national surveillance. Differences between studies in reported case numbers and geographic distribution may reflect variation in study periods, case ascertainment, referral patterns, diagnostic capacity, and access to specialized care. Further prospective surveillance, molecular characterization of clinical isolates, environmental studies, and improved recognition of the disease among healthcare professionals would help clarify the epidemiology of mycetoma in Iran.

Table 2 summarizes the Iranian case reports and case series identified in this review.

**Table 2:** Mycetoma cases and studies in Iran (1972–2026).

Study (year)	Location	Cases	Sex (M/F)	Age range	Occupation	Affected site	Etiological agents	Mycetoma type	Key findings
Asgari and Alilou (1972) [28]	Behshahr, Amol, Qazvin, Gorgan, Abadan, Babol, Miyaneh	8	5/3	25–50	3 Housewives, 3 farmers, 1 cook, 1 worker	Foot, knee, sole, forehead, leg	*Streptomyces madurae* (2), *Nocardia caviae* (4), *Madurella mycetomi* (1), *Allescheria boydii* (1)	6 Actinomycetoma, 2 Eumycetoma	First report in Iran; experimental production in mice; bone involvement in 3 cases
Griffiths et al. (1975) [32]	Southern Iran (Shiraz)	7	7/0	20–60	Not specified	Foot	*Actinomyces israelii* (4), *Nocardia asteroides* (1), *Actinomadura madurae* (1), *Allescheria boydii* (1)	5 Actinomycetoma, 2 Eumycetoma	First report from southern Iran; duration 3–15 years; bone changes in 4 patients
Ebrahimzadeh (1976) [40]	Khuzestan	1	1/0	52	Farmer	Foot	*Nocardia* spp.	Actinomycetoma	Single case from Khuzestan
Ravaghi and Aflatouni (1978) [41]	Northern Iran	6	5/1	Not specified	5 Farmers	Foot (most)	*Nocardia asteroides*	Actinomycetoma	All 6 cases were actinomycetoma
Nematian (1984) [42]	Mazandaran, Gilan, Arak, Bushehr, Zanjan, Qom	13	7/6	9–60 (Mean 40)	7 Farmers, 4 housewives, 1 merchant, 1 unemployed	Foot (12), thigh (1)	*Actinomadura madurae* (4), *Actinomyces israelii* (3), *Pseudallescheria boydii* (2), *Streptomyces somaliensis* (2), *Aspergillus fumigatus* (1)	11 Actinomycetoma, 2 Eumycetoma	Thesis on subcutaneous mycotic infections in Iran
Ghahari (1984) [43]	Khuzestan	2	2/0	24, 41	Farmer, driver	Foot, thigh	*Nocardia caviae*, *Madurella mycetomatis*	1 Actinomycetoma, 1 Eumycetoma	Case reports from Khuzestan
Ghahary et al. (1988) [31]	Iran (farmer)	1	1/0	24	Farmer	Right foot sole	*Madurella mycetomi*	Eumycetoma	Progressive disease leading to amputation after 6 years; early debridement failed
Moghaddami and Valikhani (1988) [44]	Iran	1	0/1	56	Housewife	Arm	*Pseudallescheria boydii*	Eumycetoma	Treated with ketoconazole
Moghaddami and Kordbachae (1989) [45]	Gilan, Babol, Zanjan, Gorgan, Bandar Abbas, Ghaemshahr, Kerman, Hamedan, Rasht, Tehran, Lorestan	13	8/5	20–65	6 Farmers, 2 housewives, military, plumber	Foot, ankle, leg, elbow, finger, arm	*Nocardia asteroides* (6), *Actinomadura madurae* (4), *Pseudallescheria boydii* (2)	12 Actinomycetoma, 1 Eumycetoma	Most comprehensive early case series
Shyban (1989) [46]	Isfahan, Khazar Kenar	3	3/0	23, 33, 52	3 Farmers	Foot	*Nocardia asteroides*	Actinomycetoma	All 3 cases from farmers
Khosravi and Emami (1991) [47]	Tehran	2	Not specified	24 (1 Case)	Not specified	Arm, foot	*Nocardia asteroides*	Actinomycetoma	Cases of cutaneous and subcutaneous nocardiosis
Moghaddami et al. (1991) [35]	Gilan & Mazandaran	Environmental study	N/A	N/A	N/A	N/A	*Streptomyces griseus* (31), *Nocardia asteroides* (8), *Actinomadura madura* (8), *Pseudallescheria boydii* (9), *Sporothrix schenckii* (10)	Environmental	First isolation of etiologic agents of mycetoma and sporotrichosis from nature in Iran
Forozesh et al. (1993) [48]	Tehran, Mazandaran, Isfahan	4	2/2	33, 38, 42	2 Housewives, 1 hardware seller, 1 military	Knee, foot, arm, elbow	*Nocardia caviae* (4)	Actinomycetoma	All 4 cases from N. caviae
Zarei Mahmoudabadi (1995) [49]	Khuzestan	1	1/0	50	Farmer	Right foot	*Nocardia asteroides*	Actinomycetoma	Case report from Ahwaz
Zarrin and Zarei Mahmoudabadi (1998) [50]	Khuzestan	2	1/1	42, 56	Farmer, employee	Left foot	*Nocardia brasiliensis*	Actinomycetoma	First report of *N. brasiliensis* in Khuzestan
Nourollohi et al. (1999) [51]	Isfahan	1	1/0	54	Unemployed	Buttock	Not specified	Not specified	Case report from Isfahan
Yaghoobi et al. (1999) [37]	Khuzestan	4	2/2	31, 42, 52, 56	Housewife, farmer	Right hand, left foot (3)	*Nocardia* spp., *Streptomyces* spp., *Actinomyces* spp.	Actinomycetoma	2 Cases with osteomyelitis and lytic lesions
Azimi et al. (2000) [52]	Urmia	1	1/0	71	Farmer	Left ankle	*Nocardia brasiliensis*	Actinomycetoma	Case from West Azarbayjan
Fata et al. (2000) [53]	Khorasan	9	6/3	29–67	5 Farmers, 2 housewives, 1 self‐employed, 1 unknown	Sole, sole and dorsum, leg	*Nocardia* spp. (5), *Actinomadura* (3), *Fusarium* (1)	8 Actinomycetoma, 1 Eumycetoma	Study over past decade in Imam Reza Hospital
Hashemi and Gerami (2001) [39]	Bandar Abbas	1	0/1	25	Not specified	Ankle	*Aspergillus flavus*	Eumycetoma	First report of *A. flavus* mycetoma in Iran
Emami et al. (2002) [54]	Isfahan	1	1/0	48	Farmer	Buttock	*Paecilomyces* spp.	Eumycetoma	Verrucous carcinoma overriding eumycetoma
Ziaei and Azarkar (2003) [55]	Birjand	1	1/0	32	Military	Left leg	*Nocardia* spp.	Actinomycetoma	With osteomyelitis
Shoar et al. (2003) [56]	Behshahr	1	1/0	33	Driver	Palm	*Nocardia asteroides*	Actinomycetoma	Treated successfully with co‐trimoxazole and streptomycin
Shamsoddin et al. (2004) [57]	Kerman	2	2/0	38, 41	Baker	Right foot dorsum, right foot	Not specified	Actinomycetoma	Both cases with osteoporosis, lytic lesions, and bone expansion
Enshaieh et al. (2006) [58]	Isfahan	1	0/1	40	Not specified	Disseminated subcutaneous nodules	*Pseudallescheria boydii*	Eumycetoma	Atopic patient with disseminated nodules
Zarei Mahmoudabadi and Yaghoobi (2007) [59]	Khuzestan	1	0/1	60	Housewife	Left foot	*Actinomadura madura* (presumed)	Actinomycetoma	20‐Year history; culture negative; reviewed 69 cases from Iran
Hashemi et al. (2008) [30]	Iran (nationwide)	62	41/21	18–65 (Peak 40–50)	28 Farmers (45.2%)	Pedal (49), extra pedal (13)	*Actinomadura madurae* (11), *Pseudallescheria boydii* (9), *Nocardia asteroides* (8), *Nocardia caviae* (5)	42 Actinomycetoma, 20 Eumycetoma	Retrospective study 1972–2005; ratio 42:20
Zarei Mahmoudabadi and Zarrin (2008) [29]	Iran (nationwide)	76	2:1 Ratio	9–71 (Peak 31–50, 61%)	50.8% Farmers, 24.6% housewives	Foot (66.7%), leg (11.7%), arm (8.3%)	*Nocardia asteroides* (25%), *Actinomadura madura* (25%), *Nocardia caviae* (16.1%), *Allescheria boydii* (13.4%)	84.5% Actinomycetoma, 15.5% Eumycetoma	Comprehensive review 1972–2005; most cases from North (39.5%) and South (25%)
Khodavaisy et al. (2010) [6]	Iran (nationwide)	90	66.3% M, 33.7% F	31–50 (60%)	44.4% Farmers, 22.2% housewives	Foot (73.8%), leg (4.8%), arm (4.8%)	*Actinomadura madurae* (23.5%), *Nocardia asteroides* (20.6%), *Nocardia caviae* (13.2%), *Pseudallescheria boydii* (10.3%)	85.3% Actinomycetoma, 14.6% Eumycetoma	Review 1972–2009; Mazandaran highest prevalence (59.4%) in North
Golsha et al. (2009) [60]	Gorgan, Minoodasht	2	2/0	39, 44	Road worker, unknown	Hand, foot	Not specified	Actinomycetoma	Case reports from Golestan province
Bassiri‐Jahromi (2014) [5]	Iran (nationwide)	35	22/13 (5:3)	14–80 (> 40 Years: 68.5%)	28.6% Housewives, 20% farmers	Foot (62.8%), hand (25.7%), face, trunk	*Actinomyces* spp. (15), *Nocardia* spp. (4), *Aspergillus* spp. (3), *Pseudallescheria boydii* (3), *Fusarium* (2)	54.3% Actinomycetoma, 45.7% Eumycetoma	16‐Year study (1994–2009); 28.6% had underlying medical problems
Kamali Sarvestani et al. (2022) [36]	Khuzestan	1	1/0	38	Farmer	Right foot	*Aspergillus flavus*	Eumycetoma	Diabetic patient; voriconazole treatment successful; molecular identification
Layagh Mirhosseyni et al. (2026) [38]	Iran	1	1/0	85	Farmer	Left hand	*Actinomycetes* (PCR confirmed)	Actinomycetoma	Atypical presentation without sinus tracts; hand involvement in elderly

### 2.7 Etiology of Mycetoma

More than 70 species of fungi and bacteria have been associated with mycetoma, and the number continues to expand as molecular methods improve species identification. The disease is divided into two major forms: actinomycetoma, caused by aerobic filamentous bacteria, and eumycetoma, caused by true fungi [3]. Actinomycetoma is caused by several genera, with *Nocardia brasiliensis* being particularly important in Mexico and parts of South America and *Actinomadura madurae* occurring in many endemic regions, including Iran. Other recognized agents include *Actinomadura pelletieri* and *Streptomyces somaliensis*, as well as less frequently reported *Nocardia* species such as *N. asteroides*, *N. farcinica*, and *N. nova*. The appearance of bacterial grains varies among species, but they are generally smaller and softer than fungal grains and may be white, yellow, or reddish [61].

Eumycetoma is caused by a diverse group of filamentous fungi. *M. mycetomatis* is the predominant agent globally and is particularly common in Africa. The Iranian literature shows a different pattern, with *Pseudallescheria boydii* (currently classified as *Scedosporium apiospermum* in its asexual form) among the principal reported fungal agents. Other organisms described in Iranian and international cases include *Madurella grisea*, *Acremonium* species, *Leptosphaeria senegalensis*, *Exophiala jeanselmei*, *Curvularia*, *Fusarium*, *Aspergillus flavus*, *Neohyphomyces*, and *Phoma* species [2]. Fungal grains are typically larger than bacterial grains and may be black, brown, white, or pale depending on the organism.

This geographic difference in dominant species is noteworthy. Differences in climate, soil characteristics, environmental exposure, and the distribution of fungal species could potentially contribute to this geographic pattern, but the available Iranian data do not allow these factors to be distinguished. Environmental studies specifically designed to compare the distribution of causative fungi across Iranian regions would be needed to investigate this hypothesis.

The clinical distribution of infection is addressed separately in the Sections 2.3 and 2.5. In the largest Iranian series restricted to laboratory‐confirmed cases from 1994 to 2009 (*n* = 35), the foot was involved in 65.7% of patients, followed by the hand (25.7%), while the trunk, face, and buttocks each accounted for approximately 3% of cases [5]. A history of preceding trauma was documented in 28.6% of patients [6]. These findings are consistent with traumatic inoculation as the principal route of infection, particularly among people with frequent contact with soil and plant material. Table 3 outlines key characteristics, causative agents, and clinical features of eumycetoma and actinomycetoma [3, 62].

**Table 3:** Characteristics of the major causative microorganisms and clinical features of eumycetoma and actinomycetoma.

Characteristic	Fungi (eumycetoma)	Bacteria (actinomycetoma)
Pathogenesis	Slow	Rapid
Gross appearance	Single or multiple nodules with clear margin, rare, or few sinuses.	Diffuse lesions without clear margin, multiple sinuses, and discharge of grains.
Body region involved [63]	Majority on foot (70%), hand (10%); rest other body parts	Most on foot (60%), followed by trunk (back, chest), arms, forearms, legs, knees, thighs, hands, shoulders, abdominal wall
Colors of grains [64]	Black: *Madurella mycetomatis*, *Trematosphaeria grisea*, *Leptospheria senegalensis*, *Curvularia lunata*, *Phytophthora parasitica*. White: *Acremonium falciforme*, *A. kiliense*, *A. recifei*, *Cylindrocarpon cyanescens*, *Scedosporium apiospermum*. White/pale: *Aspergillus nidulans*. Green/pale: *Aspergillus flavus*	White–yellow or pink: *Actinomadura madurae* Yellow–brown: *Streptomyces somaliensis* Red: *Actinomadura pelletieri* White–yellow: *Actinomyces israelii*, *Nocardia caviae*, *Nocardia farcinica* White: *Nocardia asteroides*, *Nocardia brasiliensis*, *Nocardia transvalensis* Cream: *Nocardia dassonvillei*
Bone invasion	Rare or late involvement	Rapid invasion
MRI and ultrasonography [65]	Small round hyperintense circles with central hypointense dots in hypointense matrix (“dot‐in‐circle” sign)	Multiple small round hyperintense circles with central hypointense dots in matrix
Standard treatment	Surgical excision plus antifungal therapy	Antibiotics
General prognosis and treatment response	Slower to respond; cure typically requires 12 months or more of therapy combined with surgery, and relapse after apparently complete treatment is well described, particularly with extensive bone involvement	More favorable overall response to medical therapy alone, though treatment courses of many months are still typical and relapse can occur if therapy is interrupted early.

### 2.8 Pathogenesis

Mycetoma develops following traumatic inoculation of environmental microorganisms into the subcutaneous tissues. The causative organisms include aerobic filamentous bacteria, such as *Nocardia* and *Actinomadura*, in actinomycetoma and a range of fungi, including *M. mycetomatis*, in eumycetoma. Minor injuries caused by thorns, splinters, or other contaminated materials can provide an entry point, particularly among people with frequent contact with soil and vegetation. Following inoculation, the organisms aggregate within characteristic grains composed of microbial filaments or hyphae embedded in an extracellular matrix. The grain structure contributes to persistence by limiting effective interaction with host immune cells and reducing the accessibility of organisms to antimicrobial treatment [66].

The grain matrix is therefore an important feature of mycetoma pathogenesis. It contains host‐derived material, including fibrin and polysaccharides, together with components produced by the microorganisms. Although the structure has some features comparable to a microbial biofilm, the two should not be considered identical. The matrix can impede phagocytosis and may reduce penetration of antimicrobial agents into the deeper parts of the grain. These properties probably contribute to the chronic nature of the infection and the limited response to medical therapy in established disease. In eumycetoma, the persistence of compact grains within fibrotic lesions is one of the factors underlying the frequent need for surgical excision in addition to prolonged antifungal treatment [66, 67].

The organisms responsible for mycetoma are widely distributed in the environment, particularly in soil and plant material. Contact with thorns and other vegetation is thought to be an important route of inoculation, especially among agricultural workers who frequently work barefoot. Several fungal pathogens have mechanisms that may enhance persistence in host tissues. For example, *M. mycetomatis* can produce melanin and metal‐binding molecules that may contribute to resistance against host defenses and environmental stress. Host recognition of the inoculated organisms activates innate immune responses, but these responses are generally insufficient to eliminate the infection and instead contribute to the chronic inflammatory reaction [2].

Clinically, the initial lesion is usually a small, painless nodule that gradually enlarges and may develop into abscesses and draining sinuses containing grains. Neutrophils accumulate around the grains and contribute to the formation of microabscesses, while macrophages and other inflammatory cells participate in the subsequent granulomatous response. Cytokines including TNF‐α, IL‐1β, IL‐6, and IFN‐γ have been implicated in the inflammatory response to mycetoma. Persistent inflammation, together with repeated tissue injury and fibrosis, can eventually result in extension into deeper tissues, osteomyelitis, and bone destruction [67].

The two forms share this general pathogenic sequence but differ in their clinical behavior and tissue response. Actinomycetoma generally progresses more rapidly and is more likely to extend into deeper tissues and bone, whereas eumycetoma often follows a slower course characterized by localized expansion and increasing fibrosis. Their grains also differ morphologically. Actinomycetoma grains are generally smaller and consist of bacterial filaments, whereas eumycetoma grains contain septate fungal hyphae and are often larger and pigmented, depending on the causative species [68].

Both forms induce innate immune responses involving neutrophils, macrophages, and granuloma formation, but differences in the balance of cellular and humoral responses have been described. Some studies suggest greater Th2‐associated activity in eumycetoma, whereas actinomycetoma may induce a more mixed Th1/Th2 response. These differences may contribute to the distinct clinical behavior of the two forms, although the immunological mechanisms underlying this divergence are not completely defined. Host susceptibility is likely to involve genetic as well as environmental factors. STAT1 is of particular interest because gain‐of‐function mutations affecting STAT1 signaling can impair IL‐12/IL‐23‐dependent immune pathways and increase susceptibility to chronic fungal infections, most notably chronic mucocutaneous candidiasis [69]. However, STAT1 mutations have not, to our knowledge, been specifically investigated in eumycetoma cohorts. Evidence for genetic susceptibility to mycetoma has been examined separately [70], but the relevance of the STAT1 pathway to mycetoma remains a hypothesis rather than an established disease‐specific mechanism.

Neutrophils are among the first immune cells recruited to the site of infection. They interact with grains, release antimicrobial molecules, and contribute to the formation of microabscesses. However, the organization of microorganisms within the grain and surrounding matrix can limit effective clearance. Macrophages subsequently participate in removal of cellular debris and grain material and contribute to the continuing inflammatory response through cytokine production [70]. Persistent recruitment of these cells, together with ineffective microbial clearance, helps explain the chronic inflammation and progressive tissue destruction characteristic of mycetoma.

### 2.9 Clinical Features

Mycetoma most commonly affects the lower limbs, particularly the foot, followed by the hands and upper limbs. Involvement of the head, neck, chest, shoulder, and other anatomical sites is less frequent. Extrapedal disease can occasionally involve the abdominal wall, face, paranasal sinuses, orbit, eyelid, or genital region, and bilateral limb involvement has also been reported. The clinical distribution may vary according to the causative organism and the site of traumatic inoculation [4].

The disease usually begins in the skin or subcutaneous tissue as a painless, firm swelling. Over time, the lesion enlarges and develops abscesses and draining sinuses containing grains. Although the classic triad of swelling, sinus formation, and grain‐containing discharge is characteristic, not all patients present with all three features. Some may initially have a small papule, nodule, cystic swelling, or localized induration without obvious sinus formation, which can delay recognition [22].

Following inoculation, the initial lesion may remain small for an extended period before gradually enlarging. As infection progresses, pustules and abscesses may form and rupture, producing purulent discharge containing grains. Multiple sinuses can develop and become interconnected through involvement of the underlying fascia. Active and partially healed sinuses may therefore coexist within the same lesion. The overlying skin may become thickened, tense, smooth, or shiny and may show areas of hypo‐ or hyperpigmentation. In advanced disease, the lesion becomes increasingly fibrotic and adherent to deeper tissues [67].

Grains are aggregates of the causative organisms embedded within an extracellular matrix and are an important diagnostic feature. Their size, consistency, and color vary according to the organism. Black grains are strongly suggestive of eumycetoma caused by pigmented fungi, whereas red, pink, white, or yellow grains may occur with different actinomycetoma and eumycetoma agents. Grain color can therefore provide a useful preliminary clue, but it should not be regarded as sufficient for species identification [71].

Despite the extent of tissue involvement, mycetoma is characteristically painless or only mildly painful during much of its course. Pain may develop with secondary bacterial infection, extensive inflammation, pressure on surrounding structures, or deeper tissue involvement. Consequently, the relative absence of pain despite progressive swelling and tissue destruction can contribute to delayed presentation and diagnosis [22].

Untreated infection generally remains localized but progressively extends along tissue planes and may eventually involve fascia, muscle, tendons, and bone. Osseous involvement can result in osteomyelitis and destructive bone lesions and is associated with more advanced disease and greater treatment difficulty. Involvement of the spine and other unusual anatomical sites has occasionally been reported. Nerve and tendon involvement is less common and generally occurs in advanced disease [72].

Regional lymph nodes may show mild enlargement, although marked lymphadenopathy should raise suspicion of secondary infection or lymphatic involvement. Lymphatic extension can occasionally produce nodular lesions along lymphatic channels, particularly in advanced actinomycetoma. Hematogenous dissemination is uncommon but has been reported. Although the two major forms can be clinically indistinguishable, actinomycetoma generally progresses more rapidly and tends to produce more extensive tissue and bone destruction, whereas eumycetoma often develops more slowly and remains relatively localized for longer periods [73].

A useful radiological finding is the “dot‐in‐circle” sign, described on MRI and, with appropriate imaging techniques, on high‐resolution ultrasonography. The sign consists of a central low‐signal or hypoechoic focus corresponding to a grain, surrounded by a higher‐signal or hyperechoic inflammatory and fibrous tissue rim. Although particularly well described in eumycetoma, similar appearances may occur in actinomycetoma. The sign is highly suggestive but not completely specific for mycetoma and should therefore be interpreted alongside clinical and microbiological findings [65].

No universally validated staging system currently exists for mycetoma. For practical treatment planning, however, disease can be described according to its extent [1]: localized disease confined to the skin and subcutaneous tissue without evidence of bone involvement [2]; locoregional disease with fascial extension or early periosteal or osseous involvement; and [3] advanced disease with extensive bone destruction, joint involvement, or spread across multiple anatomical compartments. This framework is descriptive rather than a validated clinical staging system and is used here only to support the diagnostic and treatment approach discussed below [74, 75, 76].

Because mycetoma is uncommon even in endemic settings, it can be mistaken for other chronic granulomatous, suppurative, or neoplastic conditions. This diagnostic problem may be particularly relevant in Iran, where clinicians may have limited experience with the disease [77]. Cutaneous and soft‐tissue tuberculosis can produce chronic swelling and sinus formation but usually lacks the characteristic grains and has a different histopathological profile [78, 79]. Chromoblastomycosis may present with chronic verrucous lesions resembling atypical eumycetoma but is characterized histologically by muriform or sclerotic bodies rather than mycetoma grains [80, 81]. Other important differential diagnoses include botryomycosis, sporotrichosis, chronic bacterial osteomyelitis, soft‐tissue sarcoma, and, in appropriate epidemiological settings, cutaneous leishmaniasis [82]. Demonstration of grains by direct examination remains one of the most useful initial steps in evaluating a suspected case. However, because grain morphology and color do not reliably establish species or always distinguish actinomycetoma from eumycetoma, microscopy should be followed by culture, histopathology, and molecular identification when available. This stepwise approach forms the basis of the diagnostic algorithm proposed in the following section.

### 2.10 Diagnosis

Diagnosis of mycetoma requires integration of clinical findings with examination of grains and, when necessary, microbiological, histopathological, molecular, and imaging investigations. No single method is sufficient in every case. Direct examination of grains from sinus discharge is often the first laboratory step. A 10% potassium hydroxide (KOH) preparation can rapidly demonstrate fungal hyphae and grain morphology and is particularly useful for the presumptive recognition of eumycetoma, although its sensitivity and specificity are limited. Gram staining may demonstrate the fine bacterial filaments characteristic of actinomycetoma, whereas fungal hyphae can be visualized with appropriate fungal stains. Ziehl–Neelsen staining has a limited role because most mycetoma‐associated actinomycetes are not strongly acid‐fast [7].

Fine‐needle aspiration cytology (FNAC) and biopsy can provide material for cytological and histopathological examination. Staining with hematoxylin and eosin (H&E), periodic acid–Schiff (PAS), and, where appropriate, Gram staining can demonstrate grains and the surrounding inflammatory response. PAS is particularly useful for highlighting fungal hyphae, while bacterial grains may be better characterized using routine histology together with appropriate bacterial stains. Histopathology can therefore support the diagnosis and help distinguish mycetoma from other chronic granulomatous or suppurative lesions, although it may not provide reliable species‐level identification [3].

Culture remains an important method for recovering the causative organism and can provide material for subsequent identification and antimicrobial susceptibility testing. However, culture may require prolonged incubation and can be affected by prior antimicrobial or antifungal treatment, inadequate sampling, and the slow growth of some organisms. Morphological identification of cultured isolates may also be insufficient for species‐level assignment. Molecular methods, including conventional PCR, real‐time PCR, sequencing‐based approaches, and isothermal amplification techniques such as loop‐mediated isothermal amplification (LAMP), can improve identification when conventional methods are inconclusive. Their availability, cost, laboratory infrastructure, and degree of validation remain important limitations, particularly outside specialized or reference laboratories [83].

The WHO, in collaboration with the Foundation for Innovative New Diagnostics (FIND) and other partners, has developed Target Product Profiles (TPPs) for mycetoma diagnostics. These TPPs define desired characteristics for two priority diagnostic approaches: a test capable of distinguishing eumycetoma from actinomycetoma to support initial treatment decisions, and a confirmatory test capable of identifying the causative species closer to the point of patient care. The TPP framework addresses characteristics including diagnostic performance, turnaround time, usability, and cost, with the aim of supporting diagnostic access in settings where conventional laboratory facilities are limited [65]. The Iranian diagnostic pathway has not, to our knowledge, been formally evaluated against these TPP criteria. Such an assessment could help identify specific gaps in diagnostic capacity and inform the development of a practical referral and testing network in Iran [84].

Imaging complements laboratory diagnosis by defining the anatomical extent of disease and identifying deeper tissue or bone involvement. Plain radiography can demonstrate advanced osseous destruction, while ultrasonography may identify characteristic hyperechoic foci corresponding to grains and surrounding inflammatory tissue. Magnetic resonance imaging (MRI) provides greater detail of soft‐tissue, fascial, and bone involvement and can be particularly useful for treatment planning. The dot‐in‐circle sign on MRI and, in appropriate settings, high‐resolution ultrasonography is highly suggestive of mycetoma, although it should be interpreted in conjunction with clinical and laboratory findings [7].

Table 4 compares the principal diagnostic modalities according to their diagnostic role, strengths, limitations, approximate turnaround time, and suitability for use in resource‐constrained settings. The reported performance ranges are illustrative rather than estimates derived from a single Iranian cohort, because no head‐to‐head study evaluating all modalities in the same Iranian population was identified.

**Table 4:** Comparison of the principal diagnostic modalities for mycetoma.

Method	Distinguishes eumycetoma vs. actinomycetoma	Species‐level identification	Approximate turnaround	Relative cost	Field/district‐hospital feasibility
Clinical examination alone	Suggestive only	No	Immediate	Minimal	High, but low specificity
Direct microscopy (KOH, Gram, Ziehl–Neelsen) of grains	Yes, in most cases	No (genus‐level at best)	Same day	Low	High
Fine‐needle aspiration cytology	Yes	No	Same day to 48 h	Low	High
Histopathology (H&E, PAS)	Yes	Usually no	Days	Low–moderate	Moderate (requires pathology service)
Culture	Yes	Yes, if isolate sporulates/grows characteristically	1–8 Weeks	Moderate	Moderate (requires trained mycology laboratory)
PCR/sequencing (ITS, β‐tubulin)	Yes	Yes, high accuracy	1–5 Days once received	High	Low–moderate (reference laboratory)
Antifungal/antimicrobial susceptibility testing	N/A (post‐diagnosis)	N/A	1–2 Weeks	High	Low (specialized laboratory)
MRI/ultrasonography (dot‐in‐circle sign)	Suggestive only	No	Same day (where available)	High (MRI)/moderate (ultrasound)	Low (MRI)/moderate (ultrasound)

### 2.11 Treatment and Recovery

Mycetoma is often recognized only after the lesion has progressed, partly because the early disease is usually painless and develops slowly. Treatment therefore depends on early recognition, accurate differentiation between actinomycetoma and eumycetoma, and assessment of the extent of tissue and bone involvement. Because the two forms have different antimicrobial requirements and markedly different treatment responses, identification of the causative organism is an important component of treatment planning [85]. Because response varies according to the causative organism, Table 5 summarizes the principal mycetoma‐associated genera and species and maps them to commonly used first‐line and alternative treatment options. The treatment recommendations should be interpreted in conjunction with species identification, disease extent, susceptibility data where available, and the clinical context rather than as rigid organism‐specific regimens.

**Table 5:** Pathogen‐directed treatment mapping for mycetoma.

Organism	Type	First‐line treatment	Alternative/second‐line	Notes
*Nocardia* spp. (*N. asteroides, N. brasiliensis, N. caviae*)	Actinomycetoma	Trimethoprim–sulfamethoxazole ± amikacin	Amoxicillin‐clavulanate; carbapenems (imipenem/meropenem); linezolid	Leading Iranian actinomycetoma agents alongside *A. madurae*
*Actinomadura madurae*	Actinomycetoma	Trimethoprim–sulfamethoxazole ± streptomycin or dapsone	Amikacin‐containing combination regimens	Most frequently identified pathogen in Iranian series
*Actinomadura pelletieri*	Actinomycetoma	Trimethoprim–sulfamethoxazole ± amikacin	Streptomycin‐containing combinations	Less commonly reported in Iran than *A. madurae*
*Streptomyces somaliensis*	Actinomycetoma	Trimethoprim–sulfamethoxazole ± streptomycin	Amikacin‐containing combinations	Produces characteristically hard grains (clinical features)
*Actinomyces israelii*	Actinomycetoma	High‐dose penicillin or amoxicillin	Trimethoprim–sulfamethoxazole; doxycycline	Prolonged courses (weeks–months) typical of actinomycosis‐family organisms
*Madurella mycetomatis*	Eumycetoma	Itraconazole	Voriconazole; posaconazole; fosravuconazole (trial evidence, see below)	Leading global eumycetoma agent; uncommon in Iran relative to *P. boydii*
*Pseudallescheria boydii*/*Scedosporium apiospermum*	Eumycetoma	Voriconazole	Itraconazole; posaconazole	Leading Iranian eumycetoma agent; relatively resistant to amphotericin B
*Aspergillus flavus*/*A. fumigatus*	Eumycetoma	Itraconazole or voriconazole, guided by susceptibility testing where available	Posaconazole	Iranian case demonstrated voriconazole success after itraconazole failure (causative agents)
*Fusarium* spp.	Eumycetoma	Voriconazole	Posaconazole; amphotericin B (limited activity)	Relatively uncommon cause in Iranian series

### 2.12 Treatment of Actinomycetoma

Actinomycetoma generally responds better to antimicrobial therapy than eumycetoma, although treatment is prolonged and the choice of regimen depends on the causative organism, disease extent, clinical response, and drug availability. Combination therapy with trimethoprim–sulfamethoxazole (TMP–SMX) and amikacin is among the most commonly used regimens, while other antibiotic combinations have also been reported [86]. WHO similarly describes actinomycetoma as being treated primarily with combinations of antibiotics, with TMP–SMX and amikacin among the commonly used agents [87].

Alternative antimicrobial regimens have been used in patients with inadequate response, intolerance, contraindications, or organism‐specific susceptibility patterns. These include combinations involving amoxicillin–clavulanate, imipenem, or other agents, although the evidence supporting individual alternatives is considerably less extensive than that for established TMP–SMX‐based regimens. Amikacin‐containing treatment requires particular attention to renal function and cumulative toxicity, especially when prolonged therapy or repeated treatment cycles are necessary [66, 88].

Treatment is generally continued for several months and should be guided by clinical response rather than by a fixed duration alone. Resolution of swelling, sinus formation, and discharge, together with improvement in imaging findings when bone involvement is present, can help determine the adequacy of treatment. Failure to improve despite an appropriate regimen should prompt reassessment of the diagnosis, disease extent, adherence, and, where feasible, the identification and antimicrobial susceptibility of the causative organism rather than simply prolonging an ineffective regimen [89, 90].

### 2.13 Treatment of Eumycetoma

For eumycetoma, surgical removal of the bulk of the lesion is critical because antifungal agents penetrate poorly into the surrounding fibrous tissue. Debulking reduces the fungal burden and may improve the effectiveness of subsequent medical therapy. Careful excision with adequate margins, thorough inspection of cavities and deep pockets, and systematic irrigation with iodine solution and hydrogen peroxide are important to remove residual fungal hyphae and grains [91].

Surgical management broadly follows the stage of disease outlined in the Section 2.9. Localized, mobile lesions without bone involvement are generally treated with wide local excision following a period of preoperative antifungal therapy to reduce lesion size and vascularity. Locoregional disease with early bone involvement may still be managed with debridement and bone curettage combined with prolonged antifungal therapy. In advanced disease characterized by extensive bone or joint destruction, or recurrence despite adequate medical and surgical treatment, amputation may be considered as a last resort rather than a default approach, given its substantial impact on livelihood in the predominantly rural, manually working population described in Section 2.3. Preoperative antifungal therapy, typically itraconazole for 2–3 months, is generally recommended before elective surgery to reduce lesion friability and intraoperative dissemination of grains. Antifungal therapy is continued postoperatively to address residual microscopic disease [91, 92].

Ketoconazole (400–800 mg/day), once the principal treatment for eumycetoma, is now less favored because of its hepatotoxicity. Itraconazole (400 mg/day in divided doses) is currently the standard antifungal therapy, although treatment response and duration vary [92]. When used alone, itraconazole is typically administered for 12 months or longer and, when combined with surgical debulking, is generally continued for at least 3–6 months postoperatively. Periodic monitoring of liver function is warranted because of the hepatotoxicity associated with azole therapy [93]. Terbinafine (250–500 mg/day) has limited efficacy as monotherapy but may have a complementary role when combined with itraconazole. Voriconazole (400 mg/day) and posaconazole (200–800 mg/day) demonstrate strong in vitro activity, but limited clinical evidence and the need for prolonged treatment have restricted their use. Newer agents, including fosravuconazole, a prodrug of ravuconazole, and isavuconazole, have shown promising in vitro activity and are undergoing clinical evaluation. Echinocandins and amphotericin B, despite their activity against other deep fungal infections, have poor activity against *M. mycetomatis* and are rarely used. However, amphotericin B delivered through absorbable calcium sulfate beads implanted into postoperative bone defects has been associated with improved outcomes and fewer adverse effects [2, 94].

### 2.14 Newer Antifungals and Recent Trial Evidence

Fosravuconazole, an oral prodrug of ravuconazole with a long half‐life that permits once‐weekly dosing, has been evaluated in a randomized, double‐blind, phase 2 proof‐of‐concept trial involving Sudanese patients with eumycetoma, predominantly caused by *M. mycetomatis*. The trial compared two once‐weekly fosravuconazole doses with daily itraconazole; all treatment arms were combined with surgery and administered for approximately 12 months. Complete cure rates were numerically similar across the fosravuconazole and itraconazole groups. Fosravuconazole did not demonstrate superiority over itraconazole, and all three regimens were generally well tolerated, with no new safety signals identified. Nevertheless, the convenience of once‐weekly dosing may offer an adherence advantage in the resource‐limited rural settings in which many patients present [95].

Isavuconazole has likewise demonstrated promising in vitro activity against *M. mycetomatis* and other eumycetoma‐causing fungi, although clinical evidence in patients with mycetoma remains more limited than that for fosravuconazole at the time of writing [96]. Thus, these newer agents should currently be regarded as potentially more convenient or better‐tolerated alternatives to itraconazole rather than as therapies with demonstrated superior cure rates. Pending further clinical trial evidence, itraconazole in combination with surgery remains the evidence‐based standard of care (Table 6).

**Table 6:** Treatment protocols, typical dosing, duration, monitoring, and adverse effects of the agents.

Drug	Typical adult dose	Typical duration	Key monitoring	Notable adverse effects
Trimethoprim–sulfamethoxazole	8–10 mg/kg/day trimethoprim component, divided	5–12+ Months	Full blood count, renal function	Cytopenias, rash, hyperkalemia
Amikacin	15 mg/kg/day IV/IM, often in 3‐week on/2‐week off cycles	Cyclical, alongside oral therapy above	Renal function, audiometry	Nephrotoxicity, ototoxicity
Streptomycin	14 mg/kg/day IM (adjusted by renal function)	Cyclical, as part of combination therapy	Renal function, audiometry	Ototoxicity, nephrotoxicity
Dapsone	100 mg/day	Months, as combination partner	Full blood count (hemolysis risk), G6PD status where feasible	Hemolytic anemia (especially with G6PD deficiency), methemoglobinemia
Linezolid	600 mg twice daily	Weeks–months (limited by toxicity)	Full blood count (especially with courses > 2–4 weeks)	Myelosuppression, peripheral/optic neuropathy with prolonged use
Amoxicillin‐clavulanate	875/125 mg twice daily (adult)	Months	Liver enzymes	GI upset, hepatotoxicity (uncommon)
Itraconazole	400 mg/day in divided doses	12+ Months (eumycetoma)	Liver enzymes; drug interactions	Hepatotoxicity, heart failure exacerbation, GI upset
Voriconazole	400 mg/day maintenance (after loading)	Months, guided by response	Liver enzymes; visual symptoms; drug levels where available	Visual disturbance, photosensitivity, hepatotoxicity
Posaconazole	200–800 mg/day (formulation‐dependent)	Months, guided by response	Liver enzymes	GI upset, hepatotoxicity
Fosravuconazole	Once‐weekly dosing per trial protocol	~12 Months, combined with surgery in trial	Liver enzymes	Generally well tolerated in trial; no new safety signals reported
Ketoconazole	400–800 mg/day (largely superseded)	Historically months	Liver enzymes (closely)	Hepatotoxicity (reason for declining use)

Treatment in children and older adults requires additional consideration. Pediatric cases are uncommon, but have been reported in Iran. Doses should be adjusted according to body weight and treatment guided by clinical and radiological response [29]. In older patients, renal function should be assessed before amikacin‐containing treatment for actinomycetoma, and potential drug interactions should be reviewed carefully [38].

Pregnancy also requires individualized management. Systemic azole therapy should generally be avoided when treatment can safely be postponed, and decisions should be made with specialist input. For actinomycetoma, amoxicillin–clavulanate may be considered when treatment is required during pregnancy [66, 87, 88, 97].

Several Iranian patients with mycetoma had diabetes or other underlying medical conditions. In the largest laboratory‐confirmed Iranian series, 28.6% of patients had an underlying medical problem [5]. However, the effect of these conditions on treatment response has not been systematically studied in Iran. Appropriate management of comorbidities, including glycemic control in patients with diabetes, should therefore form part of routine care.

### 2.15 Pharmaceutical Development for Mycetoma

Current treatment of eumycetoma remains challenging because therapy is often prolonged and advanced disease may require surgical intervention. These limitations, together with variable treatment responses and the risk of relapse, highlight the need for new therapies that are more effective, affordable, and less dependent on surgery. Despite this need, mycetoma has received relatively little attention in antifungal drug discovery and development compared with other infectious diseases. Recent research has focused on identifying new compounds and repurposing existing antifungal agents with activity against major eumycetoma pathogens [93]. The main mechanisms of action of established and emerging antifungal therapies are summarized in Table 7.

**Table 7:** Mechanisms of action of established and emerging antifungal therapies for eumycetoma.

Drug	Clinical status	Mechanism of action	Evidence/important findings	Key reference
Itraconazole	First‐line treatment	Inhibits fungal lanosterol 14α‐demethylase (CYP51), blocking ergosterol synthesis and disrupting the fungal cell membrane	400 mg/day for 6 months before surgery, followed by ≥ 6 months after surgery. An 80% cure rate was reported in a clinical trial, whereas a larger case series reported a corrected cure rate of 67.6%.	[61, 95, 98]
Terbinafine	Alternative treatment	Inhibits squalene epoxidase, causing sterol depletion and toxic accumulation of intracellular squalene	In a small study, 500 mg twice daily for 24–48 weeks resulted in cure or clinical improvement in 80% of patients. A retrospective study reported a 55.6% cure rate when combined with surgery.	[73, 99, 100]
Posaconazole	Investigational/repurposed azole	Inhibits CYP51, interfering with ergosterol biosynthesis	Demonstrated strong activity against *M. mycetomatis*. Small clinical studies reported successful healing in most treated patients, but evidence remains limited by very small sample sizes and high cost.	[101, 102]
Ravuconazole	Investigational	Inhibits CYP51, disrupting ergosterol synthesis	Strong in vitro activity against *M. mycetomatis* and prolonged survival in infected *Galleria mellonella*.	[103, 104]
Fosravuconazole	Clinical‐stage investigational drug	Prodrug of ravuconazole; ultimately targets CYP51/ergosterol biosynthesis	Weekly 200 or 300 mg for 6 months followed by surgery and 6 months of post‐treatment. Cure rates were 85% and 65%, respectively, but neither regimen was significantly superior to itraconazole.	[95]
Olorofim	Preclinical for eumycetoma	Inhibits dihydroorotate dehydrogenase (DHODH), disrupting pyrimidine synthesis and consequently DNA/RNA and protein synthesis	Potent activity against *M. mycetomatis* and improved survival in infected larvae. Activity was lower against some other eumycetoma species. No mycetoma patients had yet been included in clinical trials.	[104, 105]
Manogepix	Preclinical	Inhibits GWT1, disrupting glycosylphosphatidylinositol (GPI)‐anchor biosynthesis and fungal mannoprotein formation	Reduced hyphal and biofilm formation in vitro. Synergy with itraconazole was observed in vitro, but the combination did not improve survival in the animal model.	[106]
Ibrexafungerp	Repurposing candidate	Inhibits β‐(1,3)‐d‐glucan synthase, impairing fungal cell‐wall β‐glucan synthesis	Demonstrated activity against *M. mycetomatis*, prolonged larval survival, and reduced grain size in an experimental model.	[107]
Fenbendazole	Preclinical	Binds β‐tubulin, destabilizing microtubules and impairing cell division and motility	IC_50_ of approximately 3 µM against *M. mycetomatis* and significantly prolonged survival in infected larvae.	[104]
Abafungin	Preclinical/research candidate	Inhibits sterol C‐24 methyltransferase, impairing ergosterol biosynthesis	Identified as an active compound with antifungal activity; evidence for eumycetoma remains preclinical.	[108]
MMV675968	Preclinical	Inhibits dihydrofolate reductase (DHFR), affecting DNA/RNA repair, replication, methylation, and cell proliferation	Identified among promising compounds from MMV screening against *M. mycetomatis*.	[109, 110]
Fenarimol analogues	Preclinical/MycetOS	Interfere with the ergosterol biosynthesis pathway	Several analogs showed activity against *M. mycetomatis* and prolonged survival in infected larvae. Activity has so far been mainly demonstrated against *M. mycetomatis*.	[109]

### 2.16 Prevention and Control

Mycetoma is not currently a globally notifiable disease, and dedicated control programs remain limited outside Sudan. Prevention is challenging because infection usually follows traumatic inoculation of environmental organisms. Nevertheless, reducing skin exposure and protecting minor injuries are practical measures, particularly for people living and working in endemic areas. Avoiding barefoot walking and using appropriate footwear remain the main preventive recommendations [111].

Protective footwear is particularly relevant for agricultural workers, herders, and others who have frequent contact with soil and vegetation. Community education should also emphasize early cleaning and care of minor wounds. Improvements in housing and sanitation may provide additional benefits, although their specific contribution to reducing mycetoma transmission has not been well established [9, 63].

The economic value of these interventions has not been adequately studied. To our knowledge, no formal cost‐effectiveness analysis of mycetoma prevention has been conducted in Iran. Protective footwear is relatively inexpensive compared with the potential costs of advanced disease, which may include prolonged antifungal treatment, repeated surgery, rehabilitation, and loss of productivity. However, evidence from other soil‐transmitted diseases cannot be directly extrapolated to mycetoma. A country‐specific cost‐effectiveness study would therefore be useful for assessing the potential benefits of footwear and wound‐care programs in Iran [112]. Early recognition is another important component of control. Community health workers can be trained to recognize persistent painless nodules, swelling, and draining sinuses and to refer suspected cases for diagnostic evaluation. Outreach clinics may also improve access to diagnosis and treatment in rural areas where specialized services are limited [111]. Earlier diagnosis is particularly important because advanced disease is more difficult to treat and is associated with greater disability.

Prevention of disability and relapse depends on appropriate treatment and follow‐up. Patients with advanced disease may require surgery, prolonged antimicrobial or antifungal therapy, and rehabilitation. Physical therapy can help preserve mobility and function in patients with permanent tissue or bone damage. Long‐term follow‐up is also important for identifying recurrent disease [85]. Several factors continue to limit effective mycetoma control, including inadequate healthcare infrastructure, limited diagnostic capacity, low awareness among healthcare workers and communities, and the prolonged nature of treatment. Stigma and disfigurement may further delay care‐seeking [9, 14].

Sudan has developed the most comprehensive model of mycetoma control, centered on the Mycetoma Research Centre and combining clinical care, training, research, and community education. Elements of this approach could be adapted elsewhere, although implementation would need to reflect local epidemiology, healthcare infrastructure, and available resources [111]. Improved surveillance is essential for defining the actual burden of mycetoma. Standardized case definitions and reporting forms would allow cases to be compared across regions and over time. Integration of mycetoma into existing surveillance systems, where feasible, could provide a more sustainable approach than establishing a separate reporting structure. A broader international registry could also help characterize geographic distribution, treatment outcomes, and relapse patterns [14].

In Iran, several relatively simple measures could improve surveillance and prevention. First, establishing mycetoma as a reportable condition and introducing a standardized case‐reporting form would help reduce the inconsistencies in case ascertainment identified in this review. At a minimum, the form could record the patient's location, age, sex, occupation, anatomical site, causative organism, treatment, and outcome. Second, mycetoma awareness could be incorporated into existing rural primary‐care and agricultural health programs, particularly in communities where agricultural exposure is common. Third, footwear and wound‐care education could initially focus on provinces where cases have been reported most frequently, including Mazandaran, Gilan, and Golestan in the north and Khuzestan, Bushehr, and Bandar Abbas in the south. Because these geographic patterns are based mainly on published case series rather than population‐based surveillance, however, targeted interventions should be reassessed as better epidemiological data become available.

### 2.17 Policies and Guidelines

The WHO and Mycetoma Research Centre recommend establishing minimum capacity standards, using preoperative and postoperative medications to reduce relapse, monitoring drug resistance, and integrating mycetoma services into public health systems across Sudan and other endemic regions. Key priorities include multidisciplinary care teams, enhancing patient adherence, and advancing antifungal research [85].

The recognition of mycetoma as a neglected tropical disease (NTD) by the World Health Organization (WHO) has increased its visibility within the global NTD agenda. Current policy approaches emphasize integrated prevention, diagnosis, treatment, patient care, and surveillance, where feasible, within existing health and NTD programs. WHO guidance also highlights the need to strengthen health‐system capacity, improve access to essential medicines and diagnostics, and support research into more effective tools for diagnosis and treatment [9]. Since the launch of the 2021–2030 NTD roadmap, WHO efforts have included the development of diagnostic Target Product Profiles, support for training and referral networks centered on the Mycetoma Research Centre in Sudan, and advocacy for greater domestic investment in endemic countries. However, publicly available reporting on national progress toward mycetoma‐specific roadmap targets remains limited, particularly for Iran, representing an important policy and surveillance gap [113].

Implementation of mycetoma policies varies considerably between endemic countries. Sudan has the most established national framework, supported by specialized clinical services, healthcare‐worker training, community education, and referral pathways linked to the Mycetoma Research Centre. In contrast, countries such as Mexico, India, and Iran have reported substantial clinical experience with mycetoma but lack similarly dedicated national control programs [111]. Because the relative contribution of actinomycetoma and eumycetoma and the distribution of causative organisms differ geographically, national strategies should be adapted to local epidemiological and healthcare contexts rather than applying a uniform model.

Clinical guidelines emphasize that accurate differentiation between actinomycetoma and eumycetoma is central to appropriate management because the two forms require fundamentally different therapeutic approaches. Actinomycetoma is generally treated with prolonged antimicrobial combinations, commonly including co‐trimoxazole with amikacin, whereas eumycetoma usually requires prolonged antifungal therapy, principally itraconazole, together with surgical excision when feasible. Management should also incorporate assessment and treatment of bone involvement, secondary bacterial infection, wound complications, and disability. Preoperative and postoperative medical therapy may be used in eumycetoma to reduce residual disease and the risk of recurrence [47, 111].

Effective policy should extend beyond clinical treatment to prevention, early detection, rehabilitation, and financial protection. Occupational‐health programs targeting agricultural workers, community education, wound‐care practices, and access to protective footwear may reduce exposure and facilitate earlier presentation. Integration of mycetoma services into existing primary‐care and NTD programs could be particularly valuable in rural settings where specialized services are limited. Inclusion of diagnosis and treatment within national health insurance or other financial‐protection mechanisms may also reduce the economic barriers associated with prolonged therapy and repeated surgery [9, 14].

### 2.18 Implications for Iran

For Iran, the evidence synthesized in this review suggests several practical priorities. First, consideration should be given to incorporating mycetoma into the national disease‐reporting system, using a standardized case definition and reporting form that records the causative organism, anatomical site, treatment, and outcome. This would address the substantial inconsistencies in case ascertainment and denominators identified across published Iranian studies and would permit meaningful monitoring of temporal and geographic trends.

Second, establishment of at least one national or regional reference laboratory with molecular diagnostic capacity would strengthen species‐level identification and surveillance. Such a laboratory could provide confirmatory testing for unusual or diagnostically difficult cases, support antimicrobial and antifungal susceptibility testing, and facilitate standardized molecular characterization of Iranian isolates. The reported Aspergillus flavus eumycetoma case, in which molecular identification and susceptibility testing informed treatment selection, illustrates the potential clinical value of this capacity.

Third, mycetoma awareness and early case detection could be incorporated into existing occupational‐health and rural primary‐care programs, particularly those serving agricultural workers. This approach would build on existing health infrastructure rather than requiring a separate vertical program and could improve recognition of chronic painless nodules, swelling, and sinus‐forming lesions at an earlier stage.

These recommendations represent priorities derived from the evidence synthesized in this review and should not be interpreted as current official policy or stated positions of the Iranian Ministry of Health and Medical Education.

Important research gaps remain. Affordable, rapid, and field‐adapted diagnostic tests are needed, particularly tools capable of distinguishing actinomycetoma from eumycetoma and, subsequently, identifying the causative species [83]. For eumycetoma, research should prioritize shorter and more effective antifungal regimens, including evaluation of newer agents and treatment strategies that could reduce dependence on extensive surgery. Operational research is also needed to determine how mycetoma diagnosis, treatment, surveillance, and follow‐up can best be integrated into routine healthcare systems in different endemic settings [14, 85].

Finally, sustained collaboration among WHO, national health authorities, clinical centers, diagnostic laboratories, and research institutions will be essential for strengthening mycetoma control. Regional centers of excellence could provide training, reference diagnostics, clinical expertise, and research infrastructure for neighboring endemic areas. Continued advocacy at global, regional, and national levels is likewise necessary to maintain visibility of mycetoma, mobilize sustainable resources, and translate emerging diagnostic and therapeutic advances into accessible patient care [9, 111].

### 2.19 Limitations

This review is narrative rather than systematic, as described in Section Methods, 2, and this approach has important implications for interpretation. Although we used a structured search strategy, study selection ultimately relied on the authors' judgment of relevance, and no formal risk‐of‐bias or study‐quality assessment was applied. These limitations are particularly relevant because the Iranian evidence base consists predominantly of retrospective case reports and small case series rather than prospective or population‐based studies. Consequently, the epidemiological estimates presented here should be interpreted as reflecting the published literature rather than the true national burden of disease.

Publication bias is another important concern. In a literature dominated by case reports and small series, unusual, severe, refractory, or diagnostically challenging cases are more likely to be published than routine cases or patients who respond uneventfully to treatment. This may consequently distort the apparent clinical spectrum, distribution of causative organisms, geographical patterns, and reported treatment outcomes.

A further limitation is the potential overlap between published Iranian datasets. Several larger nationwide reviews re‐aggregate cases reported in earlier publications rather than representing completely independent patient populations. Therefore, simply summing cases across individual reports would risk substantial double counting and overestimate the number of reported Iranian patients. We have consequently avoided presenting a single cumulative national case count and instead interpreted the available studies according to their individual designs, populations, and periods of observation.

Finally, the absence of a national surveillance system and standardized reporting framework prevents reliable estimation of incidence, prevalence, regional distribution, and temporal trends in Iran. The apparent concentration of cases in particular provinces may therefore reflect differences in exposure and pathogen ecology, referral patterns, diagnostic capacity, and publication activity rather than true differences in disease occurrence. Prospective, multicenter surveillance using standardized case definitions, laboratory confirmation, and longitudinal follow‐up will be required to establish the actual epidemiological burden and clinical outcomes of mycetoma in Iran.

### 2.20 Research Gaps

Beyond the data‐consistency issues addressed in this revision, several important gaps remain in the Iranian mycetoma literature. Many of these cannot be resolved by further analysis of published studies and will require new primary research. The effects of climate change on the geographic distribution and seasonality of mycetoma‐causing organisms in Iran remain unclear. Likewise, antimicrobial and antifungal resistance among Iranian clinical isolates has not been systematically investigated. Further studies are also needed to examine host genetic susceptibility, including the STAT1‐related and other immune pathways discussed in Section 2.8.

A One Health perspective is another area that has received little attention. Studies combining human cases with environmental sampling and, where relevant, veterinary investigations could provide a better understanding of potential reservoirs and exposure pathways. The economic burden of mycetoma is also poorly characterized, including treatment costs, loss of productivity, disability, and the consequences of prolonged disease. Similarly, the psychosocial effects of chronic disease and visible limb disfigurement, including stigma and its effects on daily and working life, have not been adequately studied in Iranian patients.

Important gaps also exist within the health system itself. It is unclear to what extent mycetoma is currently incorporated into Iran's existing programs for neglected tropical and skin diseases. In addition, there has been no comprehensive assessment of diagnostic capacity across the country, including the availability of culture, histopathology, and molecular testing at different levels of healthcare. Such information would help identify regional gaps in diagnosis and determine where reference laboratory services are most needed.

Finally, long‐term clinical outcomes remain poorly documented. The Iranian case reports and series identified in this review provide little systematic information on relapse, persistent disability, functional outcomes, or quality of life after treatment. Prospective follow‐up studies would therefore be valuable for determining treatment durability and the longer‐term consequences of the disease.

Overall, these gaps point to a need to move beyond isolated case reports toward prospective surveillance, multicenter clinical studies, environmental and One Health investigations, health‐economic research, and standardized long‐term follow‐up. This would provide a more reliable evidence base for improving diagnosis, treatment, prevention, and health‐policy planning for mycetoma in Iran.

### 2.21 Future Research Priorities

Building on the gaps identified above, several research priorities appear particularly relevant to Iran. First, prospective and standardized national case reporting is needed to improve case ascertainment and allow more reliable assessment of geographic and temporal trends. Second, a contemporary molecular epidemiological study of Iranian isolates would be valuable, using current species‐level taxonomy and PCR‐based sequencing to update the predominantly morphology‐based identification reported in earlier studies. Third, a formal diagnostic‐accuracy study should assess current Iranian diagnostic practices against the WHO Target Product Profiles described in the Section 2.10. Finally, prospective treatment studies with standardized follow‐up are needed to evaluate clinical response, relapse, disability, and longer‐term outcomes using pathogen‐directed treatment regimens.

Given the currently limited number of reported cases, mycetoma is likely to remain a relatively uncommon but potentially disabling infection in Iran. The immediate objective is therefore unlikely to be eradication, but rather earlier recognition, more reliable diagnosis, and consistent pathogen‐directed treatment. Strengthening these areas would provide a practical foundation for improving patient outcomes while generating the epidemiological and clinical evidence needed to inform future national policy.

## 3 Conclusion

Actinomycetoma is more common than eumycetoma in Iran, although the true incidence of these diseases remains unknown due to differences in case identification and clinical overlap between them. The causative organisms also diverge from the global pattern; *Actinomadura madurae* and *Nocardia* species are the predominant agents of actinomycetoma, while *Pseudallescheria boydii* is a prominent cause of eumycetoma in the country. Demographically, the disease predominantly affects middle‐aged men in rural occupations, with most cases reported from the northern and southern provinces. However, this geographic distribution may reflect referral and publication biases rather than the true epidemiological picture.

From a clinical standpoint, mycetoma should be considered when a patient presents with a chronic, painless, sinus‐forming lesion, especially when the lesion involves the foot, and there is a history of agricultural or rural exposure. Direct examination of grains remains a useful initial investigation, with culture, histopathology, and molecular testing used when available to establish the diagnosis and identify the causative organism. Treatment should then be tailored to the pathogen and extent of disease. Earlier recognition and appropriate treatment may help prevent progression to deeper tissues and bone and reduce the need for extensive surgery or amputation.

Overall, mycetoma appears to be an uncommon but potentially disabling infection in Iran. The main priorities are better case ascertainment, improved diagnostic capacity, more reliable species‐level identification, and prospective follow‐up of treatment outcomes. These measures would provide a stronger basis for understanding the true burden of mycetoma in Iran and for developing appropriate clinical and public‐health strategies.

## Author Contributions

**Shabnam Rezaei:** software, formal analysis, data curation, conceptualization. **Khadijeh Jaberi:** conceptualization, software, formal analysis, data curation. **Maryam Meskini:** data curation, investigation, writing – review and editing. **Seyed Davar Siadat:** investigation, writing – original draft. **Abolfazl Fateh:** supervision, data curation, software, formal analysis, methodology, validation, conceptualization, investigation, writing – original draft, writing – review and editing.

## Funding

The authors have nothing to report.

## Ethics Statement

The authors have nothing to report.

## Conflicts of Interest

The authors declare no conflicts of interest.

## Transparency Statement

The corresponding author affirms that this manuscript is an honest, accurate, and transparent account of the study being reported; that no important aspects of the study have been omitted; and that any discrepancies from the study as planned (and, if relevant, registered) have been explained.

## Data Availability

No datasets were generated or analyzed during the current study.
